# Mechanical Analysis of the Acute Effects of a Heavy Resistance Exercise Warm-Up on Agility Performance in Court-Sport Athletes

**DOI:** 10.2478/hukin-2013-0077

**Published:** 2013-12-31

**Authors:** Christopher J. Sole, Gavin L. Moir, Shala E. Davis, Chad A. Witmer

**Affiliations:** 1 East Tennessee State University, Center of Excellence for Sport Science and Coach Education, Johnson City, Tennessee.; 2 East Stroudsburg University, Department of Exercise Science, East Stroudsburg, Pennsylvania.

**Keywords:** change of direction, agility, warm-up, stride length, stride frequency, ground reaction force

## Abstract

The purpose of this study was to determine the acute effects of heavy resistance exercise on agility performance in court-sport athletes. Five men (age: 20.6 ± 1.9 years; body mass: 79.36 ± 11.74 kg; body height: 1.93 ± 0.09 m) and five women (age 21.2 ± 2.7 years; body mass: 65.8 ± 10.18 kg; body height 1.77 ± 0.08 m) volunteered to participate in the present study. All subjects were NCAA Division II athletes who currently participated in tennis or basketball and all had previous resistance training experience of at least one year. In a counterbalanced design, agility performance during a 10 m shuttle test was assessed following either a dynamic warm-up (DW) or heavy resistance warm-up (HRW) protocol. The HRW protocol consisted of three sets of squats at 50, 60, and 90% of 1-RM. Agility performance was captured using an eight camera motion analysis system and the mechanical variables of stride length, stride frequency, stance time, flight time, average ground reaction force, as well as agility time were recorded. No significant differences were reported for the HRW and DW protocols for any of the mechanical variables (p>0.05), although there was a trend towards the HRW protocol producing faster agility times compared to the control protocol (p = 0.074). Based on the trend towards a significant effect, as well as individual results it is possible that HRW protocols could be used as an acute method to improve agility performance in some court-sport athletes.

## Introduction

Warm-up is a general term for routines and activities commonly used by athletes immediately prior to training or competition. The primary objective of the warm-up routine (WR) is to prepare the athlete both physically, and mentally for activity. An effective WR can acutely enhance performance and potentially reduce the likelihood of injury (Baechle and Earle, 2008). Several mechanisms responsible for performance enhancing effects of WRs have been established. These mechanisms include: increased muscle and core body temperature, resulting in an improved rate of force development (Asmussen et al., 1976), improved muscular strength and power (Bergh and Ekblom, 1979), changes to the viscoelastic characteristics of musculotendinous structures (Bishop, 2003a; Enoka, 2008), the Bohr effect (i.e. enhanced oxygen delivery), and increased blood flow to working muscles (McArdle et al., 2010).

WRs may be implemented numerous ways and consist of a variety of diverse activities. The specific characteristics of the WR are dependent on the nature of the sport, as well as the experience of the athlete and practitioner (McMillian et al., 2006). However, depending on the demands of the subsequent activity not all WR activities are appropriate. For example, WRs consisting of static stretching have been shown to impair force and power production (Behm et al., 2001; Cornwell et al., 2002; Evetovich et al., 2003), as well as decrease sprint performance (Fletcher and Jones, 2004; Winchester et al., 2008). Therefore, these WRs may not be advised immediately prior to activities involving high-velocity, explosive movements. Recently, the most common form of WR used by strength and conditioning practitioners and sport coaches is the dynamic warm-up. A dynamic warm-up involves progressive, total-body moments such as repeated lunging, squatting, and sprinting. This form of active WR has been shown to be effective in eliciting modest performance enhancements in activities requiring power and agility, when compared to static stretching or no activity (McMillian et al., 2006).

The specific activities of a dynamic warm-up can vary greatly depending on the sport, athlete, and coach. However, general guidelines for designing and implementing dynamic protocols have been suggested (Bishop, 2003b; Baechle and Earle, 2008). According to these guidelines, all routines should follow a progression from general, low-intensity activity such as 5–10 minutes of jogging or skipping, then progress toward more sport specific movements performed at higher intensities. Traditionally, the overall intensity of the WR is kept low to limit the accumulation of fatigue, production of metabolites, and depletion of energy stores (Bishop, 2003). However, there exists some evidence that high-intensity activity can better augment subsequent performance (Burkett et al., 2005; Faigenbaum et al., 2006).

One form of activity that may be potentially incorporated in WRs is heavy resistance exercise. Several studies have reported performing heavy, near maximal resistance exercise acutely improves measures of performance such as power, rate of force development, loaded and unloaded countermovement jump, and leg stiffness (Young et al., 1998; Gourgoulis et al., 2003; Comyns et al., 2007; Moir et al., 2009; Witmer et al., 2010). Additionally, researchers have reported that when heavy resistance exercise was incorporated into the WR improvements were seen in straight-line sprinting (McBride et al., 2005; Rahimi, 2007; Yetter and Moir, 2008). While measures such as countermovement jump and straight-line sprinting correlate to athletic performance it is possible they may not accurately reflect the movements performed by the athlete during sport. For instance, sprinting is an integral component of many sports, but very rarely do athletes sprint in straight lines, particularly in field and court-sports where change of direction and agility is required. It is currently unknown if heavy resistance exercise is capable of acutely enhancing a quality such as agility.

Lacking a universally accepted definition in the sports science community, agility is often associated with terms such as quickness, speed and change of direction. A key determinant of performance in the field and court-sports, agility at its most rudimentary level has been defined as the ability to accelerate, explosively brake, and accelerate again (Sheppard and Young, 2006). In actuality, agility is a complex multidimensional skill that can be attributed to multiple factors. Of these factors researchers have recently discussed the importance of leg muscle qualities in agility performance, specifically strength, reactive strength, power, and stiffness (Sheppard and Young, 2006; Young and Farrow, 2006).

From the extant literature, it can be concluded that performing heavy resistance exercises may elicit performance increases in biomechanically similar movements. Measures such as power, rate of force development, and stiffness (all play key roles in agility) have been shown to be enhanced as a result. It is therefore theoretically possible that a heavy resistance exercise warm-up protocol could be used to acutely improve agility performance, an effect that holds significant implications for athletes and strength and conditioning practitioners. However such improvements have yet to be demonstrated experimentally. Therefore, the purpose of the present research was to examine the acute effects a heavy resistance exercise warm-up on agility performance in court-sport athletes.

## Material and Methods

**Figure 1:**
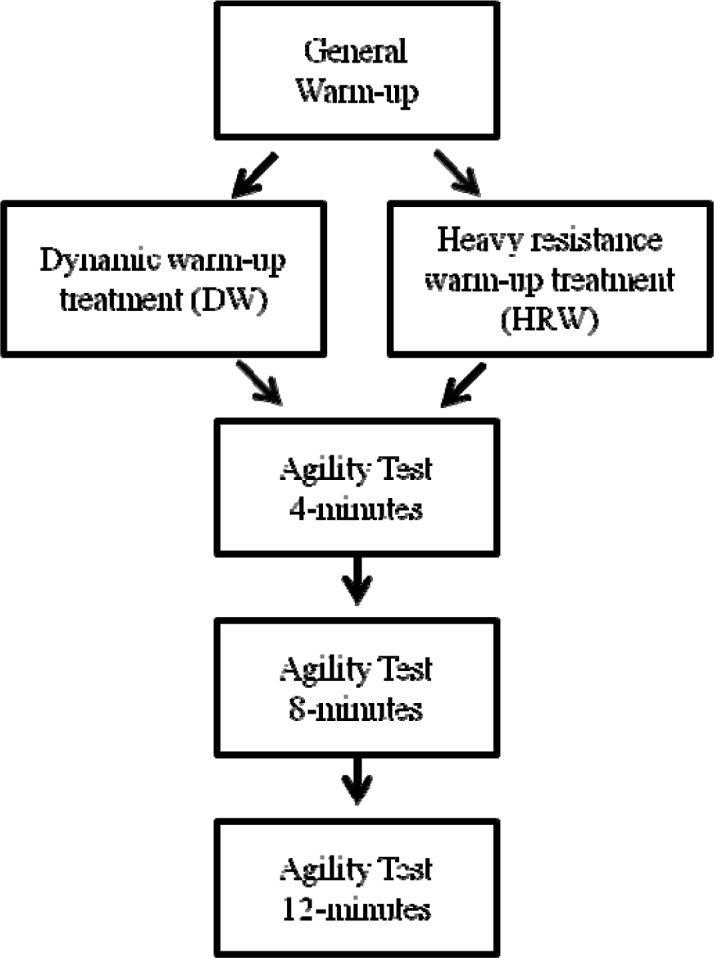
Schematic of experimental protocol.

A counterbalanced design was used to investigate the acute effects of two treatments heavy resistance warm-up [HRW], dynamic warm-up [DW]) on agility performance in court-sport athletes (Figure 1). The agility trials were performed four, eight, and twelve minutes after each treatment in order to account for any individual variations in dissipation of fatigue following treatments. Both men and women were included in the study as previous researchers have shown that there is no difference in the acute response of men and women to dynamic squat protocols (Witmer et al., 2010).

### Procedures

Subjects upon meeting the participation criteria underwent a familiarization session during which they were introduced to and allowed to practice the agility test. Following this session subjects were assessed to determine a one repetition maximum (1-RM) parallel back squat following the procedure of Baechle et al. (2008). A lift was deemed successful if the top of the thighs were parallel to the ground during the lowest point of the descent and the bar continued to move upward throughout the ascent without assistance. Spotters were used during each squat attempt, while a standard 20 kg Olympic barbell and Olympic disks (Ivanko, Reno, NV) were used during the exercise. Following the establishment of their 1-RM value, subjects were scheduled no sooner than three days later for the second testing session, which was either the HRW or the DW treatments. The subject’s experimental sessions were scheduled no sooner than two days apart to afford subjects appropriate rest. Subjects were also instructed to refrain from physical activity twenty-four hours prior to experimental sessions and to standardize their diet for the duration of the study. Prior to initiating this research all methods and protocols were reviewed and approved by the East Stroudsburg University Institutional Review Board for the Protection of Human Subjects. All subjects were informed by the investigator of all preliminary and experimental procedures and signed an informed consent form prior to participation.

### Participants

Five men (age: 20.6 ± 1.9 years; body mass: 79.36 ± 11.74 kg; body height: 1.93 ± 0.09 m) and five women (age 21.2 ± 2.7 years; body mass: 65.8 ± 10.18 kg; body height 1.77 ± 0.08 m) agreed to participate in the present study. All subjects were National Collegiate Athletic Association Division II athletes, who competed in either the sport of tennis or basketball and had at least one year of prior resistance training experience. All subjects had no prior (up to one year) lower extremity injury that could result in further harm during participation.

### Measures

#### Dynamic warm-up treatment (DW)

During the DW treatment subjects performed the general warm-up of five minutes of jogging around a 200 meter indoor track. The subjects then performed a dynamic warm-up consisting of 10 body weight squats and five walking lunges. Following a two-minute recovery the body weight squats and walking lunges were repeated. These exercises were chosen as they were deemed biomechanically similar to agility movements. Following the completion of the dynamic warm-up protocol the subjects were then led to the laboratory for agility testing.

#### Heavy resistance warm-up treatment (HRW)

The HRW treatment consisted of a general warm-up of five minutes jogging around a 200 meter indoor track, followed by three sets of parallel back squats; five squats with a load equivalent to 50% 1-RM, three squats with a load equivalent to 60% 1-RM, and three squats with a load equivalent to 90% 1-RM. A two minute recovery period was allowed between each set of squats. After the completion of the heavy resistance warm-up protocol the subjects were then led to the laboratory for agility testing.

Both warm-up treatments were designed following the guidelines suggested by Baechle et al. (2008), consisting of a general phase (5 min of jogging) and a specific phase (DW or HRW treatment). Between the two treatments, the only variation was to the specific phase where subjects performed either the DW or HRW protocol.

#### Agility shuttle test

**Figure 2:**
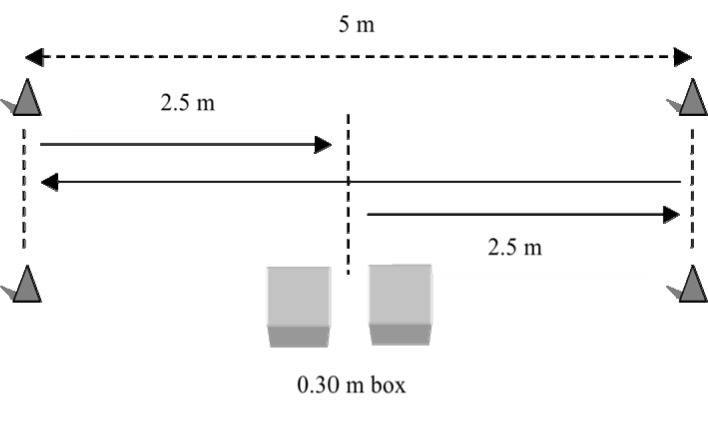
Diagram of the 10m agility shuttle test. The arrows show the path followed by a subject cutting to the right.

Subjects performed three 10 m agility shuttle tests (Figure 2). The test was modified from a standard 20 m (5m-10m-5m) shuttle test to a shorter distance due to lack of sufficient laboratory space. The agility trials were performed at four, eight, and twelve minutes post HRW or DW treatments. Times of the agility tests were recorded using a dual-beam photocell infrared timing system (Swift Performance Equipment, Lismore, Australia).

The 10 m agility shuttle test was selected due to its specificity to change of direction maneuvers used in the sports of tennis and basketball. Subjects started the test by stepping from a 0.30 m box and sprinting 2.5 m in their preferred direction. This initial sprint direction was established during the familiarization session and remained consistent throughout all tests. Subjects starting to the right direction stepped off the box to the left of the start/finish line and subjects starting to the left stepped off the box to the right of the start/finish line (Figure 2).

Stepping from a box was selected in order to maximize the role of reactive strength and stiffness at the start of the test. This also helped replicate the movements specific to actual game play e.g., landing from a shot or jump and then sprinting with changes of direction.

The reliability of the time for the modified agility shuttle test was established by using the subjects times during each of the trials following the DW treatment. The intra-class correlation was 0.96 with a 95% confidence interval (CI) = 0.88–0.99, while the coefficient of variation was 2.8% with the 95% CI = 2.1–4.4%.

An eight camera motion analysis system (Vicon™, Oxford, UK) was used to capture the maneuvers performed by the subjects during the agility shuttle test. The system sampled at 100 Hz. In order to calculate mechanical variables 39 reflective markers were placed on anatomical landmarks in accordance with the Plug-in Gait marker set. The kinematic data recorded from the markers was processed using Vicon™ Nexus 1.2 software. From the data collected using the motion analysis system the following mechanical variables were calculated: stride length, stride frequency, ground contact time, flight time, and average ground reaction force.

Stride length: Stride length (SL) was defined as the horizontal distance between the toe marker at ground contact and next ipsilateral ground contact.

Stride frequency: Stride frequency (SF) was defined as the inverse of total stride time. A stride was defined as the event between touch down and the next ipsilateral touch down selected form the vertical accelerations of the toe marker.

Stance time: Stance time (ST) was defined as the period of time between toe-down and toeoff when the foot was in contact with the ground.

Flight time: Flight time (FT) was defined as the period of time between toe-off and next ipsilateral toe-down.

Average ground reaction force: The average ground reaction force (aGRF) acting on the subject during the stance phase was calculated from the impulse-momentum relationship where the impulse of the applied force is equal to the change in momentum produced:

$$\text{Ft}={\text{mv}}_{2}-{\text{mv}}_{1}$$

where, Ft = impulse of the applied force (N·s)
mv_2_ = linear momentum at the end of force application (kg/m·s^−1^)
mv_1_ = linear momentum at the beginning of force application (kg/m·s^−1^)

The linear momentum of the subject was calculated as the product of mass and the velocity of the center of mass during the agility performance derived from the motion analysis system. Dividing the change in momentum during the stance phase by ST therefore provided the aGRF acting on the subject during stance. Both vertical (aGRFz) and horizontal (aGRFy) forces were calculated during the stance phase associated with the initial step from the 0.30 m box during the agility shuttle.

### Statistical Analysis

All statistical analyses were performed using the Statistical Package for the Social Sciences (SPSS for Windows, version 17.0, SPSS Inc., Chicago, Il). Measures of central tendency and spread of the data were represented as means and standard deviations. The fastest agility shuttle time performed following the HRW and DW treatments provided the data for the subsequent analyses. The average values for the mechanical variables of SL, SF, ST, and FT during the fastest trials were analyzed. Wilcoxon tests were used to investigate the differences in the dependent variables (agility shuttle time, SL, SF, ST, FT, aGRFz, and aGRFy) following the two treatments. Non-parametric bivariate correlations (Kendall’s Tau) were used to show the relationship between 1-RM back squat values and absolute differences in the dependent variables recorded during the agility shuttle test. The categories of Cohen (Cohen, 1988) were used to establish how meaningful the relationships were. Alpha was set at ≤ 0.05 for all analyses.

## Results

The mean 1-RM back squat for the study was 114.2 ± 35.4 kg. Table 1 displays the fastest agility times following both the HRW and DW treatments. Also shown is the time the test occurred post treatment, and the subject’s 1-RM. The Wilcoxon test revealed a trend towards the HRW protocol producing faster agility times compared to the dynamic warm-up protocol (p = 0.074). A small correlation was demonstrated between 1-RM load and the absolute change in agility shuttle time (τ = 0.26, p > 0.05).

Table 2 shows the values for SL, SF, ST, and FT during the fastest agility shuttle trials following the HRW and DW treatments. There were no significant differences reported in any of the variables as a result of performing the HRW or control protocols (p > 0.05). Trivial to moderate correlations were found between 1-RM load and the absolute changes in the stride variables (τ = 0.02 – 0.33, p > 0.05).

Table 3 shows the aGRF_z_ and aGRF_y_ during the initial step of the fastest agility shuttle trial following the HRW and DW treatments. There were no significant differences reported in any of the variables as a result of performing the HRW or dynamic warm-up protocols (p > 0.05). Small correlations were found between 1-RM load and the absolute change in aGRF_z_ (τ = 0.24, p > 0.05) and also between 1-RM load and the absolute change in aGRF_y_ (τ = 0.29, p > 0.05).

## Discussion

The purpose of this study was to determine the acute effects of a heavy resistance exercise warm-up on agility performance in court-sport athletes. This study was the first of its kind to investigate the effect of a HRW protocol on agility performance. Based on the findings of previous research it was hypothesized that the HRW treatment was likely to elicit acute performance enhancement and therefore influence subsequent agility performance through mechanism such as increased reactive strength and leg stiffness. Although group results failed to reach statistical significance, a trend towards faster agility times following the HRW treatment was observed. Seven of the ten subjects improved their agility times following the HRW protocol. Only three of the subjects produced faster agility times following the DW treatment. There are numerous mechanisms potentially responsible for the trend towards faster agility times exhibited following the HRW treatment. The possibility exists that the HRW treatment was effective in eliciting a post activation potentiation (PAP) response, an increase in the contractile ability of muscle following a bout of previous contractions (Robbins, 2005). However, it should be noted that potentiation was not measured during the present study, and identifying PAP as an underlying mechanism is purely speculative. Additionally, the HRW treatment may have resulted in increased muscle temperature, and changes in the viscoelastic behavior of the musculotendinous units compared to the DW. Again, the present study did not include assessments of these variables; therefore it is unknown whether or not these specific mechanisms were responsible for any change in performance. Future research should measure these variables, in order to better understand the nature of any acute enhancements in performance.

The reasons for the lack of significant improvements may be individual in nature and involve the contractile history of the muscle, muscle fiber type, and current phase of training. Previous research supports the effectiveness of the resistive loads used in present study’s HRW protocol in eliciting acute performance enhancements (McBride et al., 2005; Comyns et al., 2007; Rahimi, 2007). Furthermore, previous researchers have found resistive loads less than that used during the present study were insufficient in improving group performance measures (Baker, 2003; Jensen and Ebben, 2003; Comyns et al., 2007). Therefore, it would seem unlikely that the resistive loads used in the protocol were not ideal. It is important to note that although the aforementioned protocols have been shown to be effective in eliciting acute performance enhancements, these responses are highly individualized (Moir et al., 2009; Witmer et al., 2010).

Although all subjects were resistance trained by definition there was variation in maximal strength levels between subjects (Table 1). It is possible that a more homogeneous sample relative to maximal strength would have produced different results. This is in agreement with previous researchers (Young et al., 1998; Duthie et al., 2002) who concluded subject characteristics such as training status, strength levels and muscle fiber type may influence the expression of a potentiation effect following dynamic contractions with heavy resistance. However, McBride et al. (2005) concluded that the performance enhancements elicited by heavy resistance protocols appears to be independent of levels of strength and the small correlation between 1-RM load and the absolute change in agility shuttle time reported in the present study would tend to substantiate this suggestion.

Differences in the proportion of fast to slow twitch muscle fiber between subjects may be another possible factor that would explain the variation among the group results. The proportion of fast and slow twitch muscle fiber between the subjects becomes more relevant when the role of type II fibers in agility performance is considered. Subjects with predominately fast twitch muscle fiber may theoretically exhibit greater stiffness through greater reactive strength and a more rapid stretch shortening cycle. Being that stiffness is a key component of successful agility performance, subjects with predominately type II fibers would presumably produce better agility performances. Unfortunately, it was not possible to determine muscle fiber types of the subjects in the present study.

The 10 m agility shuttle test used in the present study was found to have high reliability and was designed to reflect the agility movements experienced by both tennis and basketball players. However, the dimensions and the lateral nature of the movements involved may have been more specific to the competitive movements used by the tennis players as opposed to the basketball players. This again could explain why the tennis players consistently improved following the HRW treatment. Previous researchers have emphasized the biomechanical specificity between the exercise and the performance movement when investigating the potentiating effects of heavy resistance exercise (Yetter and Moir, 2008). However, the proficiency with the movements associated with the performance test may also be a variable mediating the efficacy of heavy resistance exercise protocols.

The 10 m agility shuttle test was designed to incorporate two COD and also modified by having the subjects starting from a 0.30 m box. These movements were included in order to emphasize the role of stiffness in successful performance, something that has been identified as an important mechanical variable in agility performance (Sheppard and Young, 2006). However, agility performance is a multidimensional skill requiring the athlete to successfully combine physical components such as reactive strength and speed with perceptual elements including pattern recognition and visual scanning (Sheppard and Young, 2006; Young and Farrow, 2006). Obviously, the perceptual demands of the agility shuttle test used in the present study were limited and therefore the validity of the test could be questioned. Future research should investigate the acute effects of heavy resistance exercises on agility performance that incorporates perceptual demands.

In conclusion, there is evidence to support the theory that agility performance may be improved following a heavy resistance exercise warm-up with a trend towards significant improvements in agility times following the HRW treatment observed in the present study. Therefore, there is reason to believe that heavy resistance exercise can be incorporated into the warm-up or training routines of sports requiring high speed, explosive movements and changes of direction. However, coaches and strength and conditioning practitioners should be aware of the individualized responses to this type of treatment that may be influenced by training status, the mechanical specificity of resistance exercise relative to the performance test as well as the familiarity with the movements associated with the performance test. It may therefore be impractical to use HRW protocols with large groups of athletes and to effectively utilize HRW treatments to enhance athletic performance developing an individualized protocol for athletes may be necessary.

## Figures and Tables

**Table 1:** The fastest agility shuttle trials, the time that these were achieved following the HRW and DW treatments, and 1-RM values for each subject

Subject	HRW Treatment		DW Treatment		Agility Difference (s)	1RM(kg)

	Agility time (s)	Time post treatment (min)	Agility time (s)	Time post treatment (min)		
1	3.78	4	4.99	8	−0.31	70.0
2	2.66	12	2.77	4	−0.11	147.5
5	2.93	8	3.04	4	−0.11	110.0
4	2.95	8	3.06	8	−0.11	105.0
5	3.34	8	3.41	8	−0.07	72.5
6	2.71	8	2.78	8	−0.07	162.5
7	2.88	8	2.93	8	−0.05	95.0
8	3.39	12	3.37	4	0.02	90.0
9	2.71	8	2.65	8	0.06	170.0
10	2.09	8	2.80	8	0.1	120.0

Mean	3.02	8:24	3.09	6:48	−0.06	114.3
SD	0.36	2:16	0.43	1:56	0.11	35.5

Note: HRW - heavy resistance warm-up treatment; DW - dynamic warm-up treatment; 1-RM - one repetition

**Table 2:** Average stride length, stride frequency; stance time and flight time for each subject during the fastest agility shuttle trials following the HRW and DW treatments. Values are means ± standard deviations. Treatment

Treatment	SL(m)	SF(Hz)	ST(s)	FT(s)
HRW	2.08 ± 0.34	2.26 ± 0.30	0.20 ± 0.03	0.28 ± 0.03
DW	1.99 ± 0.32	2.27 ± 0.35	0.19 ± 0.04	0.28 ± 0.08

Note SL = stride length; SF = stride frequency; ST = stance time; FT = flight time; HRW = heavy resistance warm-up; DW = dynamic warm-up.

**Table 3:** Average vertical and horizontal ground reaction forces during the initial step performed during die fastest agility shuttle trials following the HRW and DW treatments. Values are means ± standard deviations.

Treatment	aGRFz (N)	aGRFy(N)
HRW	548 ± 145	544 ± 142
DW	535 ± 168	560 ± 172

Note: aGRFz = average vertical ground reaction force; aGRFy = average horizontal ground reaction force; COD = change of direction; HRW = heavy resistance warm-up; DW =dynamic warm-up.
